# Isolating the energetic and mechanical consequences of imposed reductions in ankle and knee flexion during gait

**DOI:** 10.1186/s12984-021-00812-8

**Published:** 2021-02-01

**Authors:** Emily M. McCain, Theresa L. Libera, Matthew E. Berno, Gregory S. Sawicki, Katherine R. Saul, Michael D. Lewek

**Affiliations:** 1 North Carolina State University, 911 Oval Drive, Raleigh, NC USA; 2 University of North Carolina at Chapel Hill, Chapel Hill, NC USA; 3 Georgia Institute of Technology, Atlanta, GA USA

**Keywords:** Biomechanics, Gait, Ankle, Knee, Metabolic cost

## Abstract

**Background:**

Weakness of ankle and knee musculature following injury or disorder results in reduced joint motion associated with metabolically expensive gait compensations to enable limb support and advancement. However, neuromechanical coupling between the ankle and knee make it difficult to discern independent roles of these restrictions in joint motion on compensatory mechanics and metabolic penalties.

**Methods:**

We sought to determine relative impacts of ankle and knee impairment on compensatory gait strategies and energetic outcomes using an unimpaired cohort (N = 15) with imposed unilateral joint range of motion restrictions as a surrogate for reduced motion resulting from gait pathology. Participants walked on a dual-belt instrumented treadmill at 0.8 m s^−1^ using a 3D printed ankle stay and a knee brace to systematically limit ankle motion (*restricted-ank)*, knee motion (*restricted-knee*), and ankle and knee motion (*restricted-a* + *k)* simultaneously. In addition, participants walked without any ankle or knee bracing (*control*) and with knee bracing worn but unrestricted (*braced).*

**Results:**

When ankle motion was restricted (*restricted-ank, restricted-a* + *k*) we observed decreased peak propulsion relative to the *braced* condition on the restricted limb. Reduced knee motion (*restricted-knee, restricted-a* + *k*) increased restricted limb circumduction relative to the *restricted-ank* condition through ipsilateral hip hiking. Interestingly, restricted limb average positive hip power increased in the *restricted-ank* condition but decreased in the *restricted-a* + *k* and *restricted-knee* conditions, suggesting that locking the knee impeded hip compensation. As expected, reduced ankle motion, either without (*restricted-ank*) or in addition to knee restriction (*restricted-a* + *k*) yielded significant increase in net metabolic rate when compared with the *braced* condition. Furthermore, the relative increase in metabolic cost was significantly larger with *restricted-a* + *k* when compared to *restricted-knee* condition.

**Conclusions:**

Our methods allowed for the reproduction of asymmetric gait characteristics including reduced propulsive symmetry and increased circumduction. The metabolic consequences bolster the potential energetic benefit of targeting ankle function during rehabilitation.

**Trial registration:**

N/A.

## Background

Acute or chronic injuries or diseases including amputations [1, 2], osteoarthritis [3–5], or stroke [6–9] can result in unilateral lower limb impairment and lead to walking that is asymmetric [10, 11], requires more positive joint work [12, 13], and is metabolically expensive [14]. Increased metabolic cost may be driven by changes in mechanical work requirements resulting from compensations for impairment of the ankle and knee [12]. For example, reduced ankle function following a stroke limits propulsion [15] which may impact swing phase mechanics [16] and correlate with decreased long-term walking function [17, 18]. Alternatively, reduced knee flexion—the cornerstone of “stiff*-*knee gait”—results in compensatory mechanisms including hip hiking and circumduction [19], which can lead to reduced walking speeds and altered joint power distribution [20, 21]. Perhaps most importantly, induced weakness at both the ankle [22] and knee [23] is reported to increase the energetic cost of walking.

Therefore, a common objective of gait interventions is to alter the underlying mechanics and reduce additional work that may be associated with metabolic penalties [24–26]. Unilateral impairments following a stroke are particularly challenging to treat because the impairment due to joint contractures and reduced muscle flexibility limit joint motion across multiple joints [27–30]. *Thus, the independent roles of ankle and knee motion on compensatory mechanics and energetic cost are difficult to discern because ankle and knee motion are interrelated.* For example, persons with stiff-knee gait also present with reductions in ankle excursion and ankle power during push-off [20] that limit knee joint velocity at toe-off and knee flexion during swing [31, 32]. Additionally, impaired limb advancement could result from either ankle or knee weakness post-stroke and lead to compensatory circumduction of the foot [33–35].

Understanding the metabolic penalties resulting from reduced motion at individual joints would provide insight into which rehabilitation or therapeutic interventions are likely to be metabolically advantageous. Changes in coordination patterns in persons post-stroke [36] make coaching a participant with hemiparesis to walk with ‘improved’ function of a joint impossible. Additionally, isolating the metabolic consequence of reduced joint function in persons post-stroke is further complicated because the changes in motor control and muscle weakness that result in joint impairment are difficult to manipulate. Instead, previous research has applied an ankle [37–39] or knee [19, 40] brace in unimpaired participants to target reductions in a single joint’s range of motion (ROM) to experimentally isolate the specific impacts of reduced ankle versus knee function. Bracing at the ankle resulted in the redistribution of power from the braced ankle to the ipsilateral and contralateral hips and an increase in metabolic cost [37]. Those authors postulated that the increase in metabolic cost resulted from the transfer of power away from the ankle joint which is suited for efficient energy storage and return through the Achilles tendon [41]. Similarly, research investigating unilateral knee bracing to simulate stiff-knee gait found increases in limb circumduction achieved through hip hiking and increased whole-body metabolic energy cost [19, 40].

Individually limiting ankle or knee ROM is known to be metabolically costly, but it is not clear which restriction is more detrimental, or how these restrictions interact. A synthesis of the literature suggests that restricting the ankle may be more metabolically costly than restricting the knee for several reasons. First, the ankle is responsible for more positive joint power than the knee during unimpaired walking [41, 42], and therefore limitations at the ankle are likely to require larger increases in positive joint power elsewhere. Second, in contrast to the ankle, during the stance phase the knee is primarily responsible for power absorption which is accomplished through negative muscle work. Because negative muscle work has a higher efficiency than positive muscle work, it is unlikely that compensations for reduced power absorptions will be as metabolically detrimental [43]. During swing, we expect impaired ankle and knee motion will both result in the inability to flex the limb and induce similar compensations and penalties. Finally, due in part to the elastic energy storage of the Achilles tendon, the ankle is a more efficient producer of positive power when compared to the knee or hip [41, 44]. Therefore, redistributing power away from the ankle to other joints is likely to increase the total cost of positive power more than redistribution from the knee to other joints [37]. Overall, with ample research suggesting the importance of the ankle in energetic efficiency, it is reasonable to hypothesize a restriction of the ankle should result in larger increases in metabolic cost than a restriction of the knee. Though previous research has begun to address metabolic impacts of restricting joints individually, no research has examined the isolated versus combined effects of reduced unilateral ankle and knee ROM on mechanical or metabolic outcomes.

The purpose of this study is to provide insight into the individual and combined effects of reduced ankle and knee ROM on gait adaptations and metabolic consequences. We used a custom 3D printed ankle stay and knee brace to isolate the impacts of reduced unilateral ankle, knee, and ankle + knee ROM on joint and limb-level compensations and the resulting metabolic consequences. Based on findings from previous literature, we hypothesized that: (h1) Limiting ankle ROM would attenuate *peak ankle power at pushoff*, reduce *peak limb propulsion* and require bilateral increases in *sagittal hip power* to compensate, (h2) Limiting knee ROM would decrease *knee flexion velocity at toe off*, impair swing limb advancement and require increased *circumduction* via ipsilateral increases in *frontal plane hip power, and* (h3) the *metabolic cost* of compensatory mechanics resulting from restricting ankle ROM would be larger than the cost of compensations from restricting knee ROM.

## Methods

### Data collection

The institutional review board (IRB) at the University of North Carolina at Chapel Hill approved all procedures, and all participants signed an IRB approved consent form prior to data collection. Data were recorded for 15 (7 M/8 F) healthy participants (age: 24.2 ± 3.0 years.; height: 1.75 ± 0.13 m; mass: 75.5 ± 15.7 kg) walking at 0.8 m s^−1^ on an instrumented split-belt treadmill (Bertec, Columbus, OH, USA). We selected this speed because it is within the range of speeds reported for persons post-stroke [26, 45], allowed ambulation with bracing restricting both the ankle and knee of simultaneously, and was sufficiently fast to allow for the detection of potential metabolic differences between conditions. Participants completed five conditions, each lasting seven minutes, including: (1) control [*control*]: no brace worn, (2) braced [*braced*]: knee brace worn but unrestricted, and *three restricted conditions*: (3) unilaterally restricted ankle [*restricted-ank*], (4) unilaterally restricted knee [*restricted-knee*], and (5) unilaterally restricted ankle + knee [*restricted-a* + *k*]. Joint bracing was achieved with a custom 3D printed ankle stay placed on the dorsum of the foot/ankle and a donJoy T-ROM knee brace (DJO Global, Inc, Vista, CA, USA). Knee bracing was worn unrestricted on both limbs in the *braced* and *restricted-ank* conditions. In the *restricted-knee* and *restricted-a* + *k* conditions, knee bracing was worn on both limbs but only restricted unilaterally. We applied lightweight ankle stays unilaterally for the *restricted-ank* and *restricted-a* + *k* conditions and removed them for all other walking conditions. The order of the *braced, restricted-ank, restricted-knee,* and *restricted-a* + *k* conditions was randomized, but the *control* condition was performed last to prevent the need for multiple marker placements per data collection. Participants wore a fall harness with no body weight support and the only instruction provided to participants was to avoid using handrails when possible. Any use of the handrails was noted by the data collection team and walking data from that timeframe was excluded from the analysis. During all conditions, we recorded rates of oxygen consumption and carbon dioxide production using a portable metabolic system (K5, Cosmed, Chicago, IL). Prior to walking trials, we collected five minutes of quiet standing to obtain baseline metabolic energy consumption. An eight-camera motion capture system (Vicon, Oxford, UK), sampling at 120 Hz, recorded the positions of 42 reflective markers attached to the pelvis and lower limb (similar marker set to [12, 26]). Marker locations in 3D space were filtered with a 6 Hz Butterworth filter in OpenSim software [46]. We recorded ground reaction forces (GRFs) recorded at 1200 Hz using the fully instrumented dual-belt treadmill. GRFs were filtered using a second order low pass Butterworth filter with a cutoff frequency of 25 Hz.

### Data processing

Data were post-processed and initial kinematic and kinetic analyses were performed in OpenSim using a full-body model [47] adapted to represent the lower limb and scaled to each participant’s anthropometry using marker locations taken during a static trial. The resulting model had six degrees of freedom describing the pelvis and six degrees of freedom per leg including three degrees of freedom at the hip, and one degree of freedom at the knee, ankle, and subtalar joints. We determined lower limb joint angles and pelvic list from filtered marker data and individual models using an inverse kinematics algorithm [48]. The inverse dynamics and analysis tools in OpenSim were used to determine joint angular velocities, moments, and powers in the sagittal and frontal plane for the hip, in the sagittal plane for the knee and ankle and in the frontal plane for the subtalar joint. We calculated joint range of motion (ROM) across the gait cycle as the difference between maximum and minimum joint angle values [37]. The max anterior GRF between 40 and 70% of gait cycle was identified as the peak propulsive force and normalized by participant mass. We calculated limb circumduction as the maximum lateral deviation from the path of progression of the foot during swing [18, 49]. Sections of anteriorly directed GRFs were integrated using the trapezium method for both limbs; propulsive symmetry was determined by dividing the contribution from the restricted limb by the sum of unrestricted and restricted limb integrated anterior GRFs. In the *braced* condition, we used the left limb in place of the restricted limb so that for any condition 50% propulsive symmetry would indicate symmetry [26, 50]. We determined joint kinematics and kinetics, pelvic list, circumduction, peak propulsion, and propulsive symmetry for 10 gait cycles, then averaged across gait cycles for each participant and trial. Pelvic list was found over the 10 gait cycles of the restricted limb for all restricted conditions, and for the left limb in the *braced* condition. Gait cycles were consecutive and selected from the last two minutes of walking in each condition by identifying and removing gait cycles bordering crossover steps and selecting the 10 consecutive gait cycles closest to the end of the two minutes from the remaining data.

We calculated average positive and negative joint mechanical power at the ankle, knee and hip as described previously [12, 26]. Briefly, the time series lower-limb joint mechanical power (watts vs. time) for each lower-limb joint was integrated in positive and negative intervals over ten gait cycles to determine mechanical work over a cycle for ankle, knee and hip (J). Gait cycle average positive and negative joint powers (W kg^−1^) were calculated by dividing the work (J) by the corresponding stride time interval (s) and normalized to each participant’s mass (kg). To isolate the impact of the brace conditions, we calculated the difference in average powers (Δaverage positive joint power) for restricted conditions relative to the *braced* condition. The total average positive joint powers were determined by summing joint powers from *both limbs* per gait cycle. Again, to isolate the impact of the brace conditions, we calculated the difference in total average positive joint power (Δtotal average positive joint power) for the restricted conditions relative to the *braced* condition.

We calculated metabolic powers from rates of oxygen consumption and carbon dioxide production during the last two minutes of each condition and quiet standing using a standard approach [51]. The net metabolic rate was determined by subtracting metabolic power of quiet standing from the metabolic power of each condition and normalizing by participant mass. To isolate the impact of the bracing conditions, we analyzed the change in metabolic rate (Δnet metabolic rate) relative to the *braced* condition. In order to evaluate the relationship between the metabolic and mechanical impacts of limiting joint ROM, the delta efficiency of positive work was computed as the linear relationship between Δnet metabolic rate and Δtotal average positive joint mechanical powers [12].

### Statistical analyses

We performed one-way (walking condition) repeated measures (participants) reduced maximum likelihood (REML) analysis using the PROC MIXED method in SAS statistical modeling software to determine if the walking condition was a significant factor for each outcome. In the absence of missing values, this method gives the same p values and multiple comparisons tests as repeated measures ANOVA. We inspected the normality of the residuals using a Q–Q plot generated by the SAS model described above. For any outcome measures without clearly discernable linear trends in the Q–Q plot we further investigated the normality of the residuals using Shapiro-Wilkes analysis using the PROC UNIVARIATE method. Only one outcome measure was not normally distributed. To remedy this, we used the PROC ROBUSTREG method is SAS to test for outliers, and after removing one outlier we performed another Shapiro Wilkes analysis to confirm normality and ran paired t-tests. For outcome measures that are presented relative to the *braced* condition (Δsagittal plane average positive hop power, Δfrontal plane average positive hip power, Δmetabolic cost, and Δtotal average positive joint power) we made three comparisons including: (1) *restricted-ank* vs *restricted-knee*, (2) *restricted-ank* vs *restricted-a* + *k*, and (3) *restricted-knee* vs *restricted-a* + *k.* For all other data, we made the following six comparisons: (1) *braced* vs *locked-ank*, (2) *braced* vs *restricted-knee*, (3) *braced* vs *restricted-a* + *k*, (4) *restricted-ank* vs *restricted-knee*, (5) *restricted-ank* vs *restricted-a* + *k*, and (6) *restricted-knee* vs *restricted-a* + *k* conditions. We corrected used a Bonferroni correction for multiple comparisons. We determined the significance of a linear correlation between using Pearson’s linear correlation coefficient only after confirming normality of the variables plotted.

## Results

We first sought to establish whether restricting joint ROMs had the intended effect on the target joints. We subsequently assessed each respective hypothesis regarding the effect of ankle and knee restriction on compensatory mechanics. Finally, we evaluated our final hypothesis regarding Δnet metabolic rate in response to imposed joint restrictions.

### Restricted limb ankle angle, velocity, and power

**Fig. 1:**
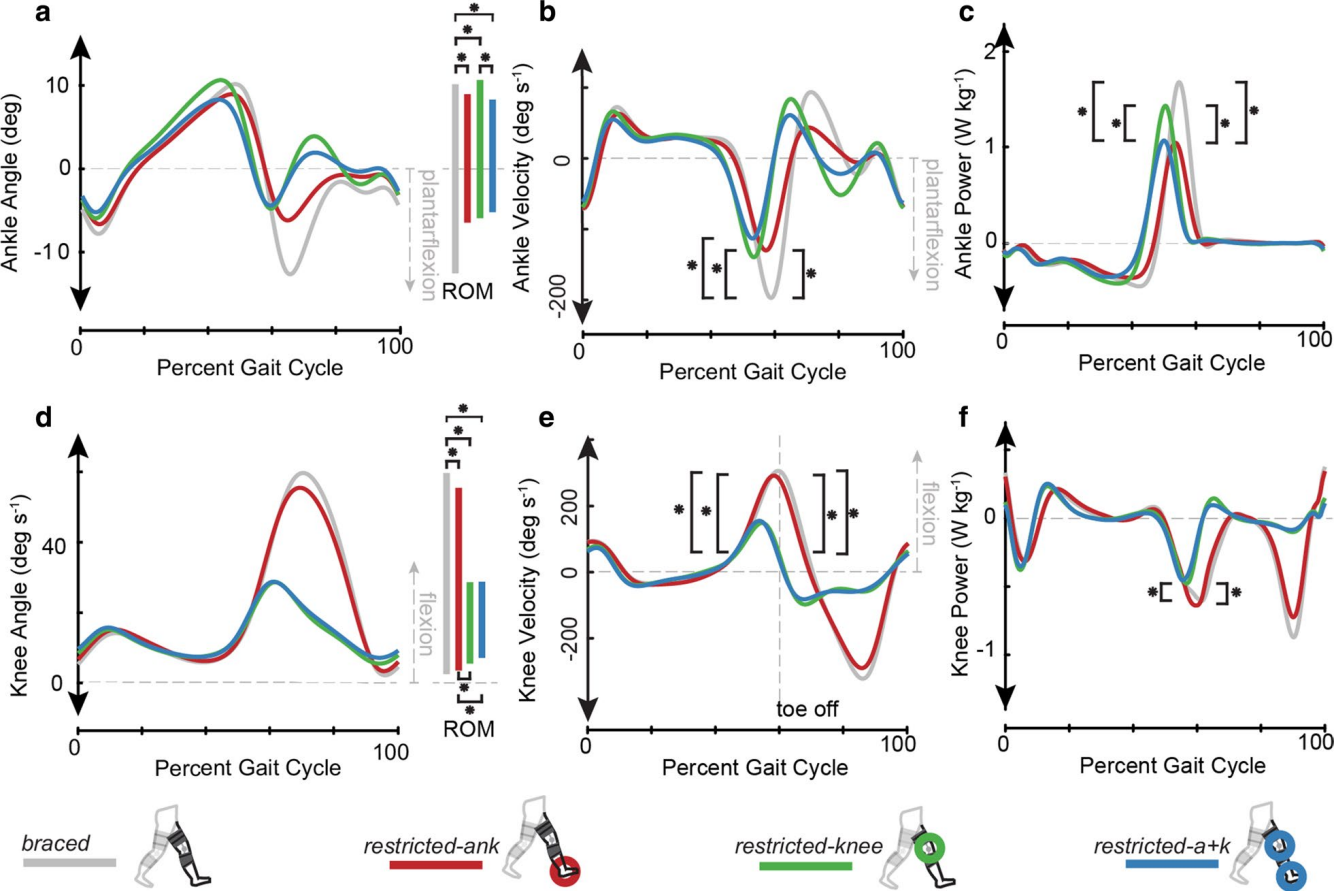
Bracing at the ankle and knee limits subject average (N = 15) joint ROM, velocity, and mechanical power. Conditions with any restriction of joint motion (*restricted-ank, restricted-knee, restricted-a* + *k)* show **a** reduced ankle ROM and **b** reduced magnitudes of peak ankle velocity during pushoff when compared to the *braced* condition. In the *restricted-a* + *k* condition, ankle ROM decreased in comparison to the *restricted-knee*. **c** Peak ankle power decreased in all conditions with ankle restriction (*restricted-ank, restricted-a* + *k)* when compared to other conditions (*braced, restricted-knee)*. **d** Knee ROM decreased in all conditions with restriction of joint motion (*restricted-ank, restricted-knee, restricted-a* + *k)* when compared to the *braced* condition, and was further reduced in conditions bracing the knee (*restricted-knee, restricted-a* + *k)* when compared to the *restricted-ank* condition. All conditions with knee bracing had **e** reduced knee joint velocity at toe off when compared to the *braced* and *restricted-ank* conditions, and the magnitude of **f** peak knee joint power absorption at pushoff was decreased in the *restricted-knee* condition when compared to *restricted-ank* and *braced* conditions. Asterisks indicate a statistically significant difference (post-hoc paired t-test with Bonferroni correction for multiple comparison, p < 0.05)

Walking condition significantly impacted restricted limb ankle range of motion (ROM), restricted limb ankle minimum velocity during pushoff, and peak restricted limb ankle power (all p < 0.001). (1): Ankle ROM (Fig. 1a, Additional file 1: Fig. S1) was significantly reduced in the *restricted-ank*, (16.9787° ± 3.59°; p < 0.0001), *restricted-knee* (17.96° ± 3.67°; p < 0.0001)*,* and *restricted-a* + *k* (14.61° ± 3.78°; p < 0.0001) conditions when compared to the *braced* (24.11° ± 5.06°) condition. We also found significant reductions in ankle ROM for the *restricted-a* + *k* condition when compared to the *restricted-knee* (p = 0.002) condition. (2) Minimum restricted limb ankle velocity during pushoff (Fig. 1b, Additional file 2: Fig. S2) was significantly reduced in the *restricted-ank*, (− 145.43 ± 43.06 deg s^−1^; p < 0.0001) *restricted-knee* (− 152.40 ± 41.55 deg s^−1^; p < 0.0001)*,* and *restricted-a* + *k* (− 130.38 ± 37.68 deg s^−1^; p < 0.0001) conditions when compared to *braced* (− 215.73 ± 46.78 deg s^−1^) condition. (3) Peak restricted limb ankle power: We observed significant reductions in peak restricted limb ankle power (Fig. 1c, Additional file 4: Fig. S4) in the *restricted-ank* (1.31 ± 0.53 W kg^−1^) when compared to the *restricted-knee (*1.66 ± 0.64 W kg^−1^; p = 0.012) or *braced* (1.94 ± 0.69 W kg^−1^; p < 0.0001) conditions. Peak restricted limb ankle power was also reduced in the *restricted-a* + *k* (1.35 ± 0.53 W kg^−1^) when compared to the *restricted-knee *(p = 0.038*)* or *braced* (p < 0.0001) conditions.

### Restricted limb knee angle, velocity, and power

Walking condition had a significant effect on restricted limb knee joint ROM (p < 0.0001), restricted limb knee flexion velocity at toe off (p < 0.0001), and restricted limb knee power absorption during pushoff (p = 0.0004). We found significant reductions in (1) restricted limb knee ROM (Fig. 1d, Additional file 1: Fig. S1) in all three braced conditions (*restricted-ank* 53.23° ± 5.66°, p = 0.01, *restricted-knee:* 4.84° ± 4.14°, p < 0.0001, *restricted-a* + *k*: 24.22° ± 5.82°; p < 0.0001) when compared to the *braced* condition (58.55° ± 3.85°). Further, reductions in restricted limb knee ROM were present in the *restricted-knee* and *restricted-a* + *k* conditions (p < 0.001) when compared to the *restricted-ank* condition. We found significant reductions in (2) restricted limb knee flexion velocity at toe off (Fig. 1e, Additional file 2: Fig. S2) in the *restricted-knee* (48.34 ± 58.88 deg s^−1^, p < 0.0001) and *restricted-a* + *k* (33.03 ± 82.86 deg s^−1^, p < 0.0001) conditions when compared to the *braced* (305.25 ± 44.16 deg s^−1^) condition. When compared to the *restricted-ank* (278.48 ± 48.19 deg s^−1^) condition, we found significant reductions in restricted limb knee flexion velocity at toe off in the *restricted-knee* (p < 0.0001) and *restricted-a* + *k* (p < 0.0001) conditions. (3) restricted limb knee power absorption (Fig. 1f, Additional File 4: fig. S4) during pushoff was larger in the *braced* (− 0.7681 ± 0.23 W kg^−1^, p = 0.020) and *restricted-ank* (− 0.825 ± 0.200 W kg^−1^, p = 0.0003) conditions when compared to the *restricted-knee* condition (− 0.6433 ± 0.18 W kg^−1^). All joint angles, velocities, moments, and powers are reported in Additional Files 1–4: Figs. S1, S2, S3, and S4, respectively.

### Peak restricted limb propulsion

**Fig. 2:**
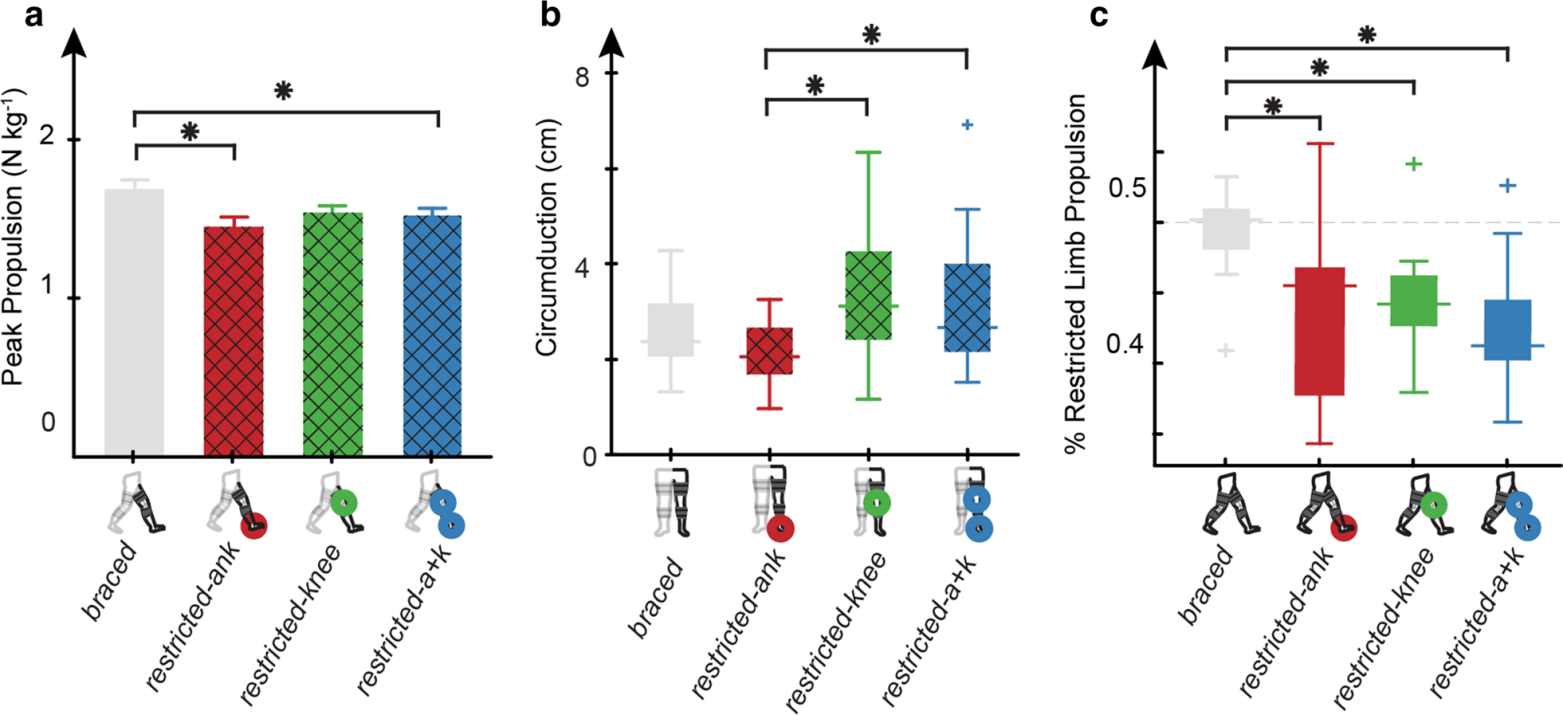
Joint level restrictions propagated to limb-level changes in peak propulsion, circumduction, and % restricted limb propulsion. Conditions with ankle restriction (*restricted-ank, restricted-a* + *k)* show decreased **a** subject averaged (N = 15) peak propulsion on the restricted limb when compared to the *braced* condition. Error bars are mean + s.e.m. Limited knee flexion in the *restricted-knee* and *restricted-a* + *k* conditions resulted in increased **b** subject averaged (N = 15) circumduction when compared to the *restricted-ank* condition. Error bars are mean ± s.d. Any restriction of joint motion (*restricted-ank, restricted-knee, restricted-a* + *k)* resulted in a reduction in **c** subject averaged (N = 15) propulsive symmetry when compared to the *braced* condition. Error bars are mean ± s.d. Black hatched lines were used for data calculated on the restricted limb in one of the three restricted conditions. Asterisks indicate a statistically significant difference (post-hoc paired t-test with Bonferroni correction for multiple comparison, p < 0.05)

Walking condition had a significant effect on peak propulsion of the restricted limb (p < 0.0001) (Fig. 2a). Post-hoc analysis revealed restricted limb peak propulsion was significantly decreased in the *restricted-ank* (1.45 ± 0.25 N kg^−1^, p = 0.0011) and *restricted-a* + *k* (*1.52* ± *0.20* N kg^−1^, p = 0.040*)* conditions when compared to the *braced* (1.68 ± 0.16 N kg^−1^) condition. Although limb propulsion decreased in the *restricted-knee* condition (1.54 ± 0.18 N kg^−1^) when compared to *braced*, this change was not significant (p = 0.094).

### Propulsive symmetry

Walking condition had a significant effect on propulsive symmetry (Fig. 2c, p < 0.0001). We found that all restricted conditions (*restricted-ank:* 44.1 ± 6.6%, p = 0.0002*; restricted-knee:* 44.4 ± 3.8%, p = 0.0006; *restricted-a* + *k:* 42.3 ± 4.43%, p < 0.0001) exhibited a reduction in the propulsive symmetry compared to the *braced* (49.5 ± 2.92%) condition.

### Restricted limb circumduction

On the restricted limb, the braced conditions had a significant effect on circumduction values (p = 0.0032). Restricted limb circumduction (Fig. 2b) was significantly higher in the *restricted-knee* (3.35 ± 1.29 cm, p = 0.005) and *restricted-a* + *k* (3.19 ± 1.50 cm, p = 0.02) conditions when compared to the *restricted-ank* (2.10 ± 0.65 cm). There was no significant difference between the *braced* condition and any of the restricted conditions. Walking condition did not have a significant effect on the unrestricted limb’s circumduction (p = 0.715).

### ΔAverage positive hip joint power

**Fig. 3:**
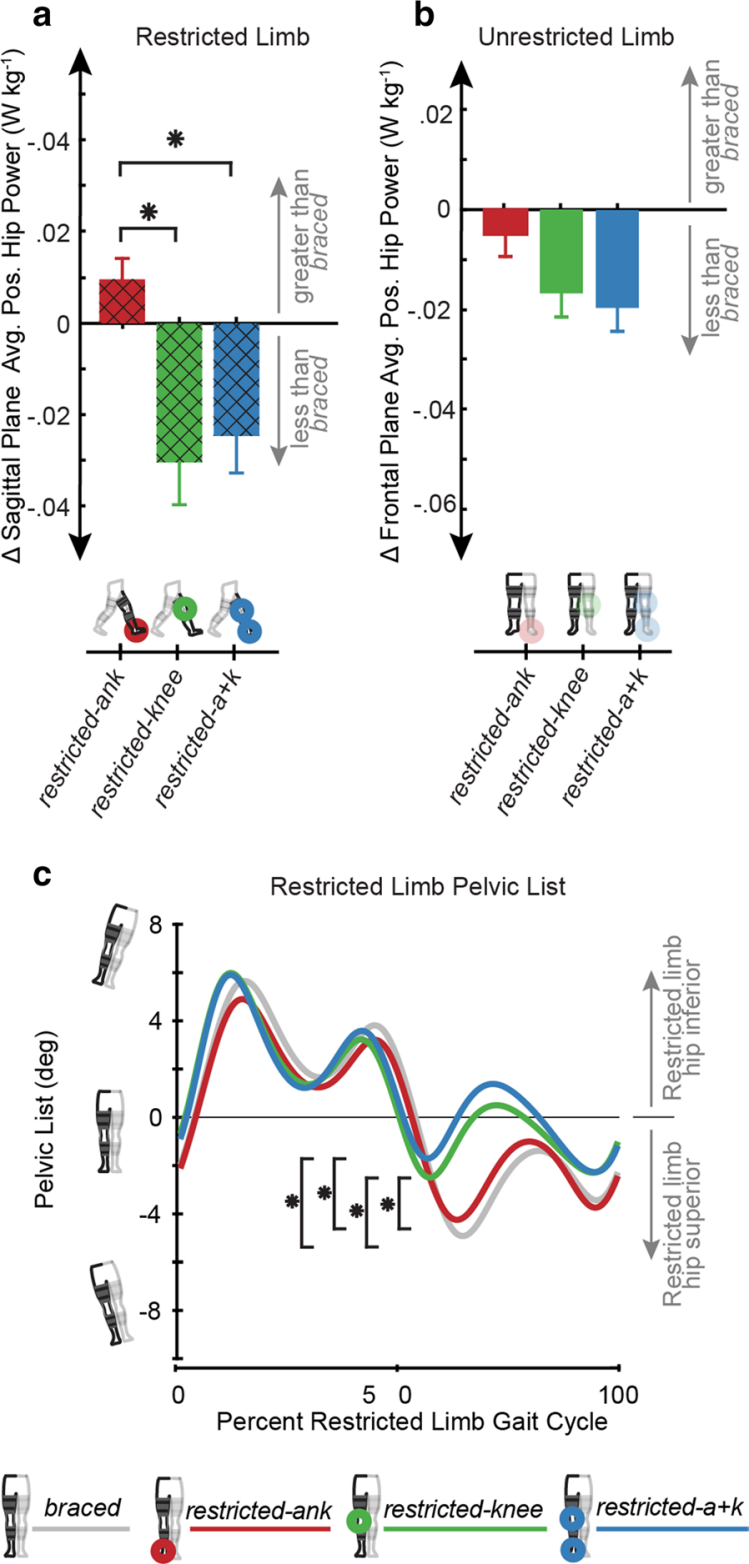
Significant impact of joint restrictions on Δsagittal plane average positive hip power and pelvic list. In the sagittal plane we see increases in **a** subject averaged (N = 15) restricted limb Δaverage positive hip powers in the *restricted-ank* condition compared to the *restricted-knee* and *restricted-a* + *k* conditions. Error bars are mean ± s.e.m. In the frontal plane of the unrestricted limb we saw a decreased **b** subject averaged (N = 15) Δaverage positive hip power values in all conditions. Error bars are mean ± s.e.m. **c** subject averaged (N = 15) pelvic list was shifted upward in the swing phase in the *restricted-knee* and *restricted-a* + *k* conditions when compared to the when the knee was immobilized to accomplish foot circumduction without increasing frontal plane hip power. Black hatched lines were used for data calculated on the restricted limb in one of the three restricted conditions. Asterisks indicate a statistically significant difference (post-hoc paired t-test with Bonferroni correction for multiple comparison, p < 0.05)

Walking condition had a significant effect (p = 0.0003) on restricted limb sagittal Δaverage positive hip power. We found significant increases in *sagittal Δaverage positive hip power* (Fig. 3a) in the *restricted-ank* (0.009 ± 0.017 W kg^−1^) condition when compared to the *restricted-knee* (-0.030 ± 0.034 W kg^−1^, p = 0.001) or the *restricted-a* + *k* (− 0.025 ± 0.031 W kg^−1^; p = 0.003) conditions. Walking condition did not have a significant effect (p = 0.83) on restricted limb *frontal Δaverage positive hip power*. Walking condition did not have a significant effect on unrestricted limb sagittal (p = 0.19) or frontal plane (p = 0.062) Δaverage positive power. While there was no significant change between conditions, the *Δaverage positive frontal plane hip power* (Fig. 3b) on the unrestricted limb was negative for all conditions.

### Pelvic list

Walking condition had a significant effect (p < 0.001) on minimum pelvic list in early swing (55–70% of gait cycle). We found a significant decrease in minimum pelvic list magnitude (Fig. 3c) during swing in the *restricted-knee* (− 2.92 ± 2.35°) condition when compared to the *braced* (− 5.30 ± 3.08°; p = 0.0008) and *restricted-ank* (− 4.18 ± 2.51°; p = 0.010) conditions. Similarly, minimum pelvic list magnitude decreased in the *restricted-a* + *k* (− 2.07 ± 2.64°) condition when compared to the *restricted-ank* (p = 0.0001) and *braced* (p < 0.0001) conditions.

### Net metabolic rate

**Fig. 4:**
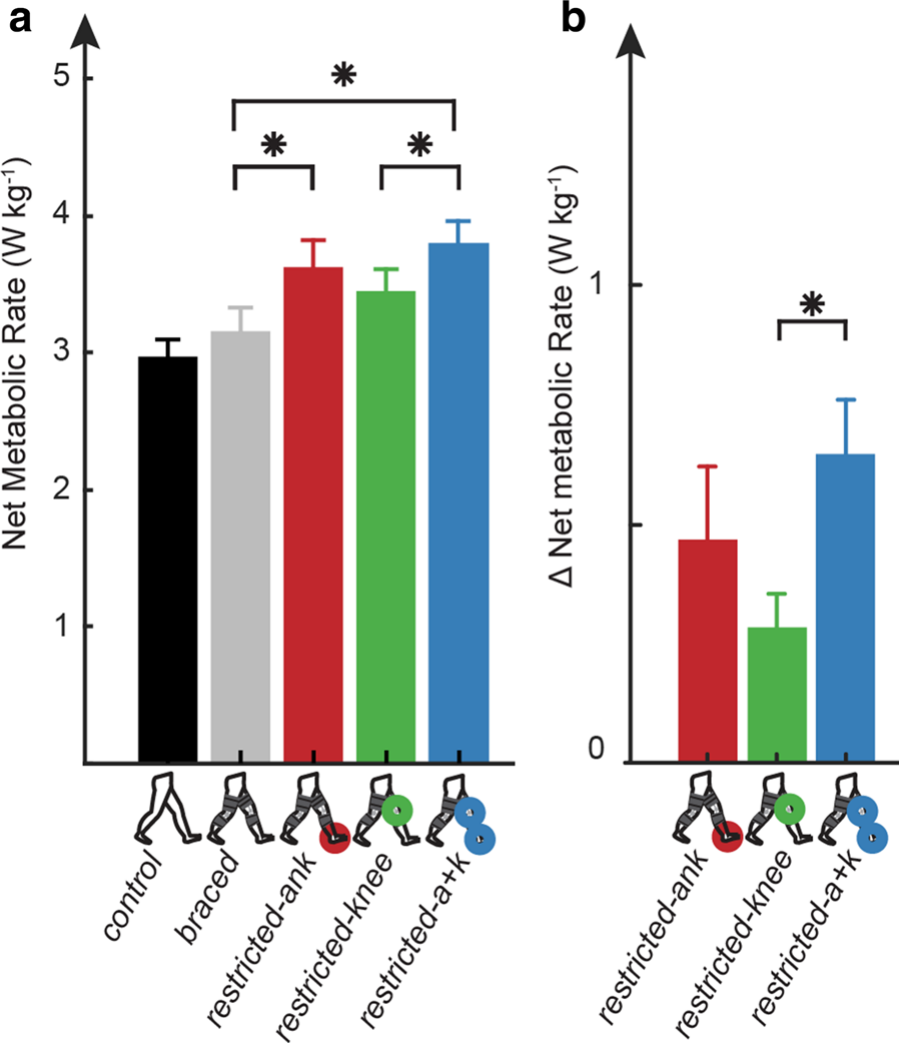
Ankle restriction increases net metabolic rate and Δnet metabolic rate. **a** The subject averaged (N = 15) net metabolic rate increased in all conditions with ankle restriction when compared to the *braced* condition, and the simultaneous restriction of the ankle and knee was more expensive than the restriction of the knee in isolation. The subject averaged (N = 15) **b** Δnet metabolic rate increased significantly in the *restricted-a* + *k* condition when compared to the *restricted-knee* condition. All **a**, **b** error bars are mean ± s.e.m. Asterisks indicate a statistically significant difference (post-hoc paired t-test with Bonferroni correction for multiple comparison, p < 0.05)

Walking condition had a significant effect on net metabolic rate (Fig. 4a, p < 0.0001). We determined the *restricted-ank* (3.59 ± 0.81 W kg^−1^, p = 0.0006) and *restricted-a* + *k* (3.77 ± 0.71 W kg^−1^, p < 0.0001) conditions were significantly more metabolically expensive than the *braced* condition (3.13 ± 0.72 W kg^−1^), and the *restricted-a* + *k* was significantly more expensive than the *restricted-knee* (3.59 ± 0.81 W kg^−1^, p = 0.0092). Walking condition also had a significant (p = 0.0018) effect on Δnet metabolic rate (Fig. 4b). ΔNet metabolic rate in the *restricted-a* + *k* (0.64 ± 0.46 W kg^−1^) condition was significantly higher than *restricted-knee* (0.28 ± 0.28 W kg^−1^, p = 0.001) condition. The Δnet metabolic rate in the *restricted-ank* (0.46 ± 0.61 W kg^−1^) condition was not significantly different from the *restricted-knee* condition (p = 0.17).

### Total average positive joint power

**Fig. 5:**
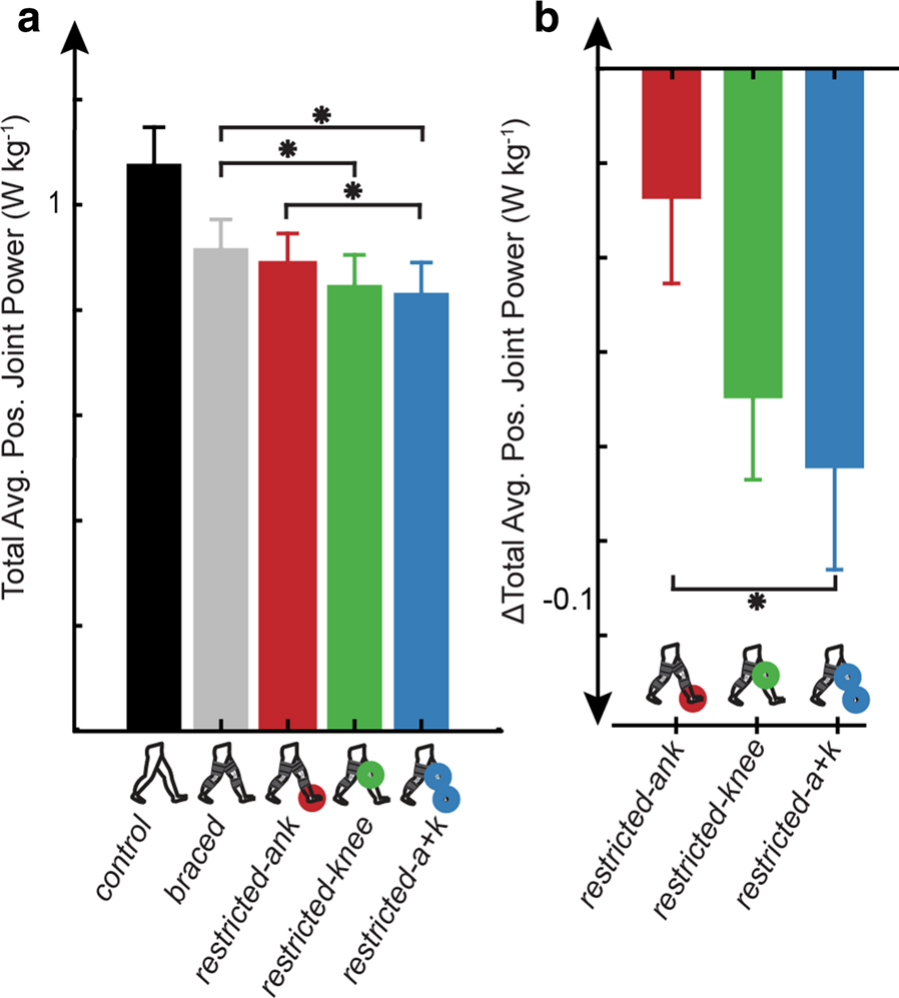
Total average positive joint power and Δtotal average positive joint power tend to decrease with joint restriction. Restriction of the knee (*restricted-knee*, *restricted-a* + *k*) resulted in reduced **a** subject averaged (N = 15) total average positive joint power compared to the *braced* condition, and the *restricted-a* + *k* condition was significantly reduced in comparison to the *restricted-ank* condition. **b** Subject averaged (N = 15) Δtotal average positive joint power in the *restricted-a* + *k* condition was significantly more negative than the Δtotal average positive joint power in the *restricted-ank* condition. All **a**, **b** error bars are mean ± s.e.m. Asterisks indicate a statistically significant difference (post-hoc paired t-test with Bonferroni correction for multiple comparison, p < 0.05)

Walking condition had a significant effect on total average positive joint power (Fig. 5a, p = 0.0008). The total average positive joint power was significantly lower in the *restricted-knee* (0.848 ± 0.22 W kg^−1^; p = 0.003) and *restricted-a* + *k* (0.832 ± 0.22 W kg^−1^; p = 0.0002) conditions when compared to the *braced* (0.917 ± 0.215 W kg^−1^) condition. Further, we found a reduction in total average positive joint powers in the *restricted-a* + *k* condition compared to the *restricted-ank* (0.89 ± 0.22 W kg^−1^, p = 0.024) condition. The distribution of positive and negative joint powers for all conditions are included in Additional file 5: Fig. S5.

### ΔTotal average positive joint power

Walking condition also had a significant effect on the Δtotal average positive joint power (Fig. 5b, p = 0.041). The Δtotal average positive joint power was significantly more negative in the *restricted-a* + *k* (− 0.085 ± 0.083 W kg^−1^; p = 0.01) condition when compared to the *restricted-ank* (− 0.028 ± 0.69 W kg^−1^) condition.

### Correlation between Δtotal average joint power and Δnet metabolic power

**Fig. 6:**
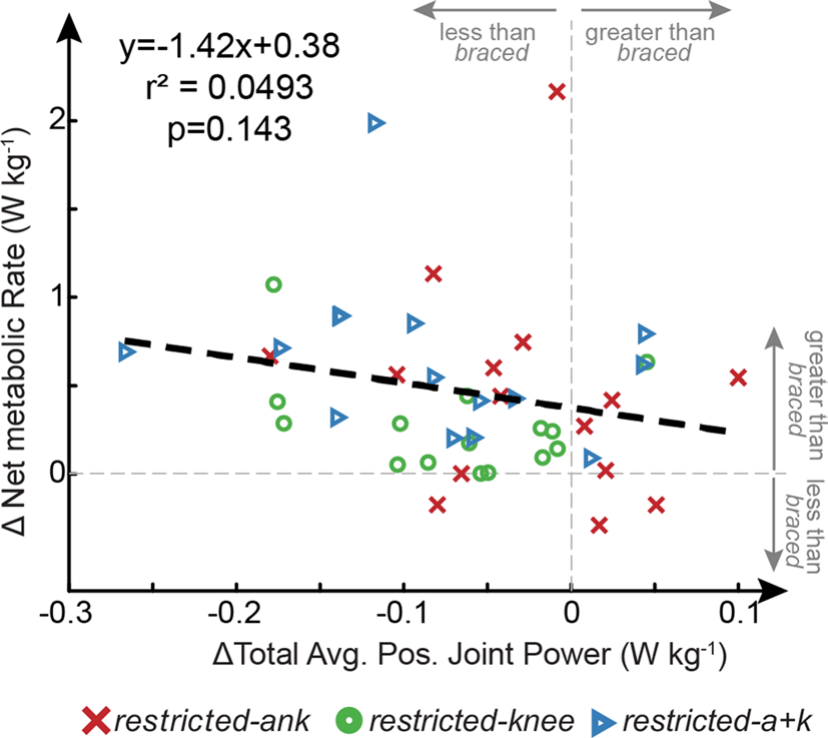
ΔTotal average positive joint power versus Δnet metabolic rate. The subject averaged (N = 15) Δnet metabolic rate and Δtotal average joint power show no statistically significant correlation and appear to have a negative correlation indicating that positive work is not an appropriate estimate of metabolic cost in atypical gait. No significant linear correlation was found using Pearson’s linear correlation coefficient

No significant correlation (p = 0.143) was found between the Δnet metabolic power and Δtotal average positive joint power (Fig. 6).

## Discussion

Our approach successfully achieved unilateral, joint-specific restrictions in range of motion (ROM) in an isolated fashion at the ankle, knee, and ankle + knee simultaneously. This framework allowed us to separate the relative impact of ankle versus knee restriction and understand their interaction on mechanical compensations and the resultant energetic penalties during walking. This research builds upon previous studies in which the ankle [37] or knee [19] were braced independently. These results can help optimize future designs of rehabilitative techniques and technology by providing insight into trade-offs of intervening at one lower-limb joint versus another.

In support of our first hypothesis, the use of our custom 3D-printed ankle stay produced a reduction in ankle ROM, which in turn attenuated peak ankle power at pushoff (Fig. 1a, c) and reduced peak restricted limb propulsion (Fig. 2a). Specifically, when the ankle was restricted, with or without locking the knee, we observed reductions in both peak ankle power and peak limb propulsion. In contrast, locking the knee did not lead to a reduction in peak propulsion (Fig. 2a), providing further evidence that ankle impairments alone may be responsible for commonly observed propulsive deficits in pathologic gait.

We hypothesized that reductions in propulsion resulting from limited ankle mobility would necessitate sagittal plane compensations at both hips; however, Δsagittal plane average positive hip power only increased on the restricted limb when comparing the restricted ankle condition to the restricted knee and combined ankle + knee conditions (Fig. 3a, b). ΔSagittal plane average positive hip power did not increase whenever the knee was restricted, suggesting that the additional restriction at the knee prevented a sagittal plane hip compensation. It is possible that the restriction at the knee made foot clearance a priority or limited the hip flexor’s capacity to initiate passive knee flexion, thereby reducing motivation for in-plane compensation.

With respect to our second hypothesis, the restriction of knee ROM, with or without the ankle restriction, contributed to an increase in circumduction when compared to only locking the ankle (Fig. 2b). Interestingly, the increases in circumduction observed in knee-restricted conditions were not significantly larger than the *braced* condition, and the circumduction values found in the *braced* condition were larger than values reported with the ankle restricted, although not significantly. We cannot attribute this finding to wearing unrestricted knee braces because knee braces were worn in all conditions except the control. It is possible that bracing the ankle and the resulting increases in sagittal plane hip power limited hip motion in the frontal plane.

We must reject part of our second hypothesis, as we did not observe the increases in frontal plane hip power that we hypothesized would facilitate circumduction of the foot (Fig. 3b). Instead, we found that participants opted to hip hike (i.e., decrease pelvic list) during restricted limb swing to enable circumduction when the knee was restricted (Fig. 3c). Interestingly, all restricted conditions had negative Δhip power in the frontal plane, indicating that any bracing reduced hip power generation when compared to the *braced* condition (Fig. 3b). This finding contrasts with the previously observed increases in frontal plane non-paretic hip power reported for persons post-stroke [12]. It is possible that the isolated bracing in our study left pelvic list as the simplest compensation for our participants, whereas individuals post-stroke typically are contending with alterations in motor control and activation in addition to stroke-induced weakness.

Our approach of restricting motion at a joint mimicked many gait characteristics of post-stroke walking (Additional Files 1–4: Figs. S1–S4). Locking the ankle resulted in reductions in ankle ROM comparable to paretic ankle ROM values reported in literature [26]. Reductions in knee joint velocity at pushoff induced by knee restriction were within the range of velocity values reported in stiff-knee literature [52]. The peak ankle powers were within the range of values seen previously in stroke survivors walking at similar speeds [26]. Propulsive symmetry decreased in all of the restricted conditions when compared to the unrestricted condition and peak restricted limb propulsion values for conditions with restricted ankle motion were within, [24, 53] but generally on the higher end of values seen in post-stroke literature [17, 53, 54]. When the knee was restricted, we observed increases in circumduction and decreases in peak knee flexion that were very similar to values reported in the literature for persons post-stroke [16, 18, 40, 49, 52, 55].

Despite our success in inducing gait characteristics common to post-stroke, our metabolic results did not support our third hypothesis that restricting the ankle would be more expensive than restricting the knee joint (Fig. 4b). Nevertheless, our results suggested an energetic impact due to ankle restriction. Specifically, our data indicated that combined restriction of the ankle and knee was more metabolically detrimental (i.e., larger positive Δmetabolic cost) than restriction of just the knee. Furthermore, all conditions that restricted the ankle (i.e., *restricted-ankle* and *restricted-a* + *k*) were more metabolically costly than the *braced* condition, suggesting that regardless of restrictions in knee ROM, any direct restriction on the ankle was metabolically detrimental. These results provide support for the potential of ankle-based rehabilitative techniques or technologies in persons post-stroke or other lower extremity joint deficits to provide a metabolic benefit [26, 56–59].

We anticipated that the increases in metabolic cost during the restricted conditions would be attributed to altered joint power requirements, consistent with findings from post-stroke gait [12, 13, 21, 60]. In particular, we expected that greater metabolic cost would be due to a combination of the concurrent transfer of power from more to less efficient joints, thereby requiring more metabolic energy to achieve the same mechanical power output and increased total average positive joint power [19, 37]. Specifically, we anticipated a bilateral increase in hip power would accompany an ankle restriction, indicating that joint power requirements were transferred from the highly efficient ankle to the less efficient hips. Instead, we only observed an increase in average positive hip power for the restricted limb in the *restricted-ank* condition when compared to the *braced* condition (Fig. 3a). Further, an increase in total positive joint powers does not appear to explain the increased energetic requirements because whereas metabolic cost tended to increase across all restricted conditions (Fig. 4), the average positive joint power tended to decrease compared to the unrestricted condition (Fig. 5). This contradicts prior work on mechanics and energetics of walking in persons post-stroke which has suggested that increases in net metabolic power are accompanied by increases in total average positive joint power without a change in the efficiency of positive mechanical work [12, 13, 21, 60]. While a decrease in total average positive joint powers between conditions with equivalent walking speed may seem counterintuitive, an increase in gait cycle duration would allow for the conservation of total joint work. Additionally, we did not observe a significant correlation between Δtotal average positive joint power and Δnet metabolic power (Fig. 6). Overall, changes in total average positive joint power were a poor indicator of changes in net metabolic power study-wide (Figs. 4, 5, 6, Additional file 5: Fig S5). Thus, in general, it need not be true that changes in metabolic cost are driven by changes in positive mechanical power under conditions with restricted joint ROM. It is possible that this discrepancy in findings is due to the inherent differences in mechanically-induced joint restrictions used here and the unilateral muscle weakness and altered muscle control present after stroke. Specifically, while our study was able to reproduce ‘stroke-like’ gait by restricting joint kinematics, we do not reproduce neural changes altering muscle-level coordination complexity [36], changes in muscle reflex coupling [61], or changes in muscular contraction efficiency [6] that exist post-stroke. These results warn that the use of positive joint power as a proxy for metabolic demand when analyzing atypical walking may be tenuous [62]. Other factors, such as muscle activation and effort, may be more relevant to mechanisms driving metabolic cost [63, 64].

There are limitations to this work that require consideration. While bracing at the ankle and knee restricted ankle excursion and knee velocity to values within the range reported for persons post-stroke, we cannot account for the neuromechanical changes that accompany a stroke (see above). We recognize that participants may have used the trunk and upper extremity to compensate for restricted lower limb motion, and the way in which the upper limb was used may also affect the lower limb mechanics reported here. If we had these data, our regression analysis of total average joint powers may be a stronger predictor of metabolic cost. As we look to generalize these results to impaired populations it is important to note that neurological injury could restrict upper limb compensations and have possible effects on measured lower limb function. Additionally, as part of a larger study examining bilateral vs. unilateral restriction, our participants’ knee braces were worn bilaterally (albeit unrestricted on one side in all conditions) and may have altered gait when compared to the unbraced *control*. We attempted to account for this limitation by comparing the restricted conditions to the *braced* condition, during which the knee braces were both worn unrestricted. Our choice to compare to the *braced* condition was made to eliminate the impact of the additional mass of the knee braces. An ankle stay was added onto the participant before the *restricted-ank* and *restricted-a* + *k* conditions and removed following the conditions so it is also possible that the added mass of the ankle stay could have impacted outcomes; however, the ankle stay was 3D printed out of PLA and weighed less than 3 oz, and therefore we do not believe the risk of mass-related impacts to be significant. While the exact amount of time needed to acclimate to unilateral bracing is unclear, we attempted to mitigate this limitation by analyzing walking trials from the last 2 min of each 7 min condition. The participants of this study were on average significantly younger than the average person post-stroke, which may impact generalizing our results to older populations. Comfortable overground walking speed in persons post-stroke can also be significantly less than 0.8 m s^−1^, the speed participants walked in this research. However, the walking speed chosen here was designed to be fast enough to challenge the walkers and elicit metabolic changes, but slow enough for them to complete the braced trials. Lastly, we cannot generalize our results to a situation where existing joint or limb limitations led participants to reduce walking speed; future research could investigate the impact of joint restriction on gait compensations and metabolic consequences across walking speeds.

## Conclusions

This work provides insight into the relative contributions of the ankle versus knee on walking mechanics and energetics to better inform how to target interventions for rehabilitation of gait post-stoke. We successfully employed ankle and knee braces to isolate the effects of limited ankle motion versus knee motion, as well as examine the combined effects of simultaneously restricted ankle and knee motion. Our approach reproduced many mechanical features of hemiparetic gait at the joint and limb levels including reduced ankle power, reduced knee velocity, reduced restricted limb peak propulsion and increased restricted limb circumduction. Unilaterally restricted ankle function induced biomechanical compensations that were particularly detrimental to metabolic demand, bolstering the argument that ankle-centric rehabilitation has the potential to improve walking energetics post-stroke. Interestingly, the large increases in metabolic cost observed with both ankle and knee restricted simultaneously were accompanied by a decrease rather than an increase in total average positive joint power relative to the *braced* condition. This result raises questions about the utility of a work-efficiency approach for understanding mechanics and energetics of gait that has atypical coordination and suggests the need to explore force or activation-based proxies for energetic demand. Finally, restricting kinematics to achieve atypical gait patterns may not capture the complicated changes in coordination that drive changes in mechanics and energetics in populations with neural impairments. Future work is warranted to understand links between *neuro*-mechanics and energetics, that is, how changes in motor coordination rather than mechanics per se, influence metabolic cost of walking.

## Supplementary Information

**Additional file 1: Fig. S1.** Subject average joint angles. Subject average (N=15) joint angles for the control, braced and all restricted conditions for the unrestricted limb (left column) and the restricted limb (right column).

**Additional file 2: Fig. S2.** Subject average joint velocities. Subject (N=15) average joint velocities for the control, braced and all restricted conditions for the unrestricted limb (left column) and the restricted limb (right column).

**Additional file 3: Fig. S3.** Subject average joint moments. Subject average (N=15) joint moments for the control, braced and all restricted conditions for the unrestricted limb (left column) and the restricted limb (right column).

**Additional file 4: Fig. S4.** Subject average joint powers. Subject average (N=15) joint powers for the control, braced and all restricted conditions for the unrestricted limb (left column) and the restricted limb (right column).

**Additional file 5: Fig. S5.** Average positive (top) and negative (bottom) joint power distribution. Sections of pie chart represent the subject averaged (N=15) ankle (white), knee (light grey), and hip (dark grey) contributions and are organized by the following columns (from left to right): 1) control, braced, restricted-ank, restricted-knee, and restricted-a+k. Average positive joint powers were summed across both limbs in the top row, and average negative joint powers were summed across both limbs on the bottom row. The distribution of positive and negative power within each limb were indicated in the pie charts in the second and third row of the figure, respectively. Note that the diameters were scaled by dividing the sum of joint contributions for each pie by the maximum sum of average positive or negative joint powers (control) and that hatch patterns were used to indicate joints restricted.

## Data Availability

The datasets used and/or analyzed during the current study are available at: http://pwp.gatech.edu/hpl/archival-data-from-publications/.

## Abbreviations

GRF: Ground reaction force
IRB: Institutional review board
ROM: Range of motion
REML: Reduced maximal likelihood
