# Supplementary figures and images for: Isolating the energetic and mechanical consequences of imposed reductions in ankle and knee flexion during gait
Source: J Neuroeng Rehabil. 2021 Feb 1;18:21. doi: 10.1186/s12984-021-00812-8 (PMC7852087; doi:10.1186/s12984-021-00812-8)

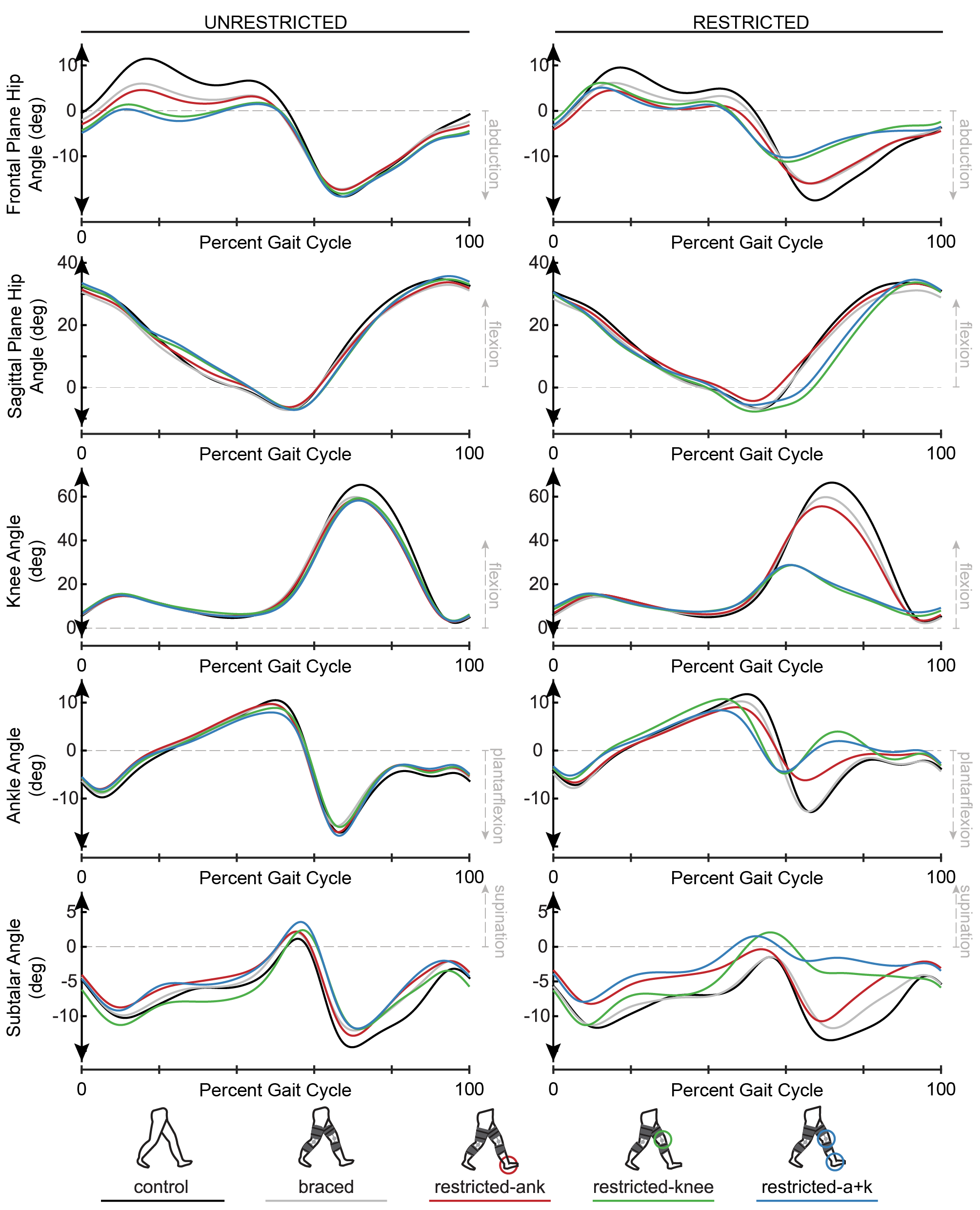

Supplement: Supplementary file 1 — Additional file 1: Fig. S1. Subject average joint angles. Subject average (N=15) joint angles for the control, braced and all restricted conditions for the unrestricted limb (left column) and the restricted limb (right column). [file 12984_2021_812_MOESM1_ESM.png]

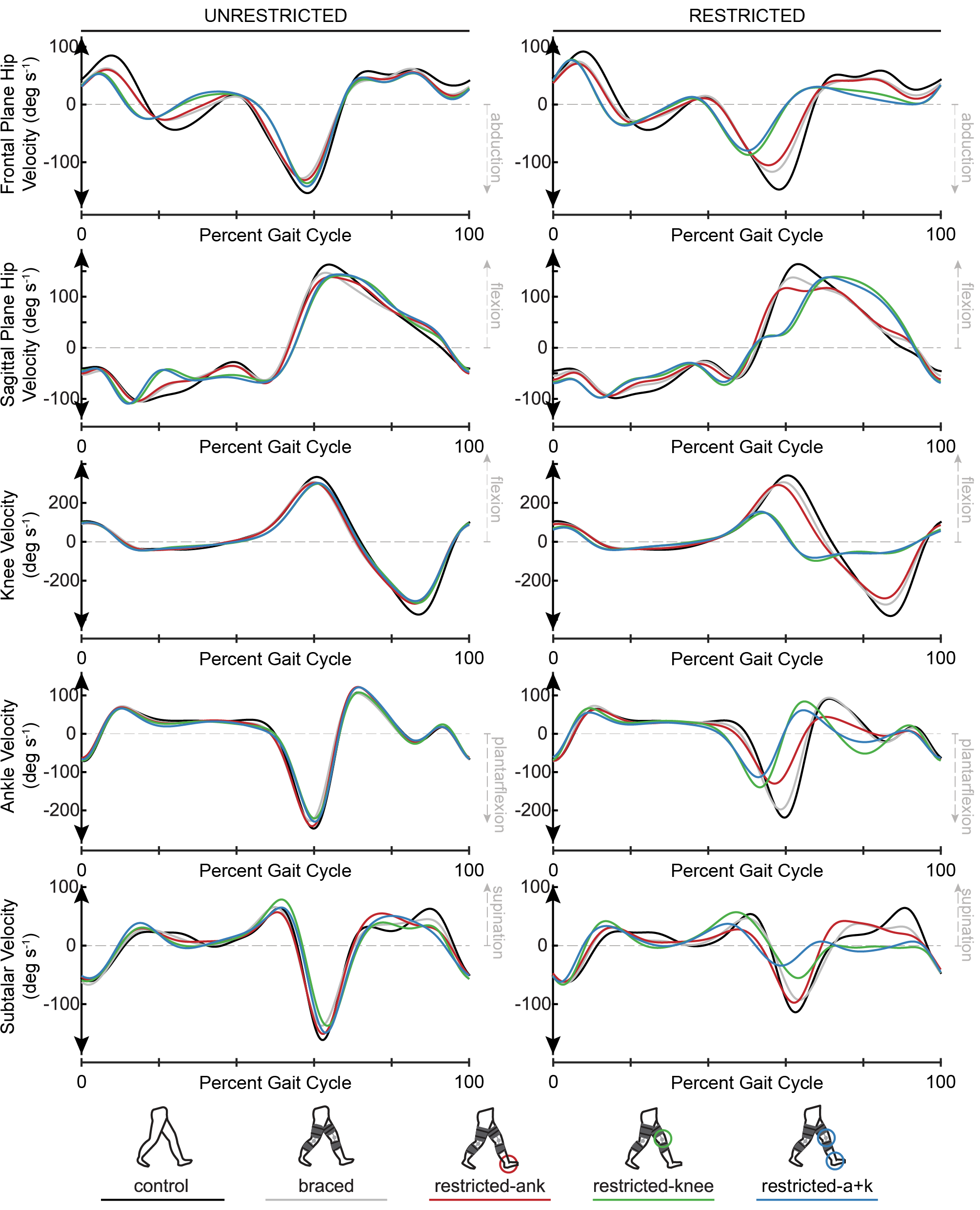

Supplement: Supplementary file 2 — Additional file 2: Fig. S2. Subject average joint velocities. Subject (N=15) average joint velocities for the control, braced and all restricted conditions for the unrestricted limb (left column) and the restricted limb (right column). [file 12984_2021_812_MOESM2_ESM.png]

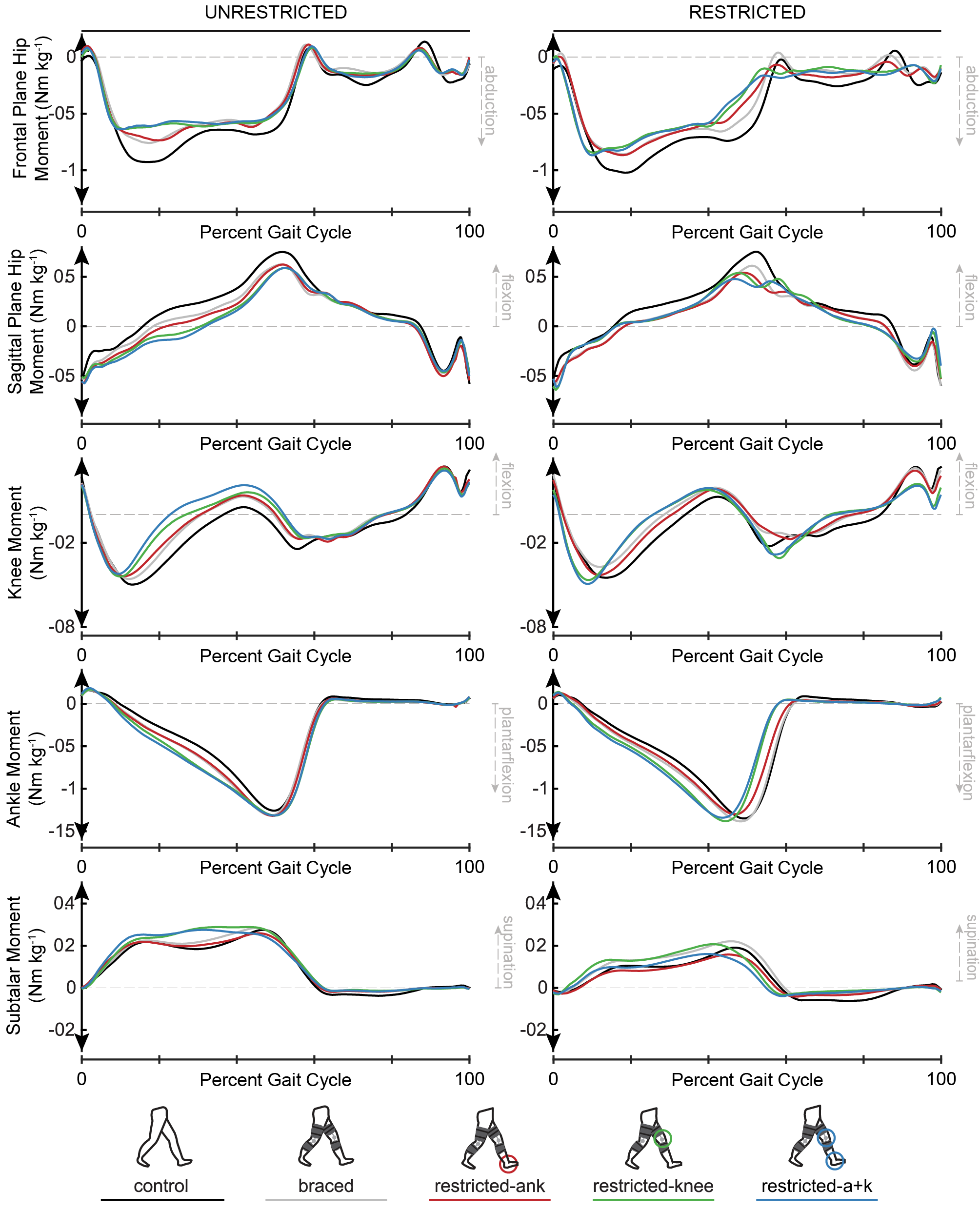

Supplement: Supplementary file 3 — Additional file 3: Fig. S3. Subject average joint moments. Subject average (N=15) joint moments for the control, braced and all restricted conditions for the unrestricted limb (left column) and the restricted limb (right column). [file 12984_2021_812_MOESM3_ESM.png]

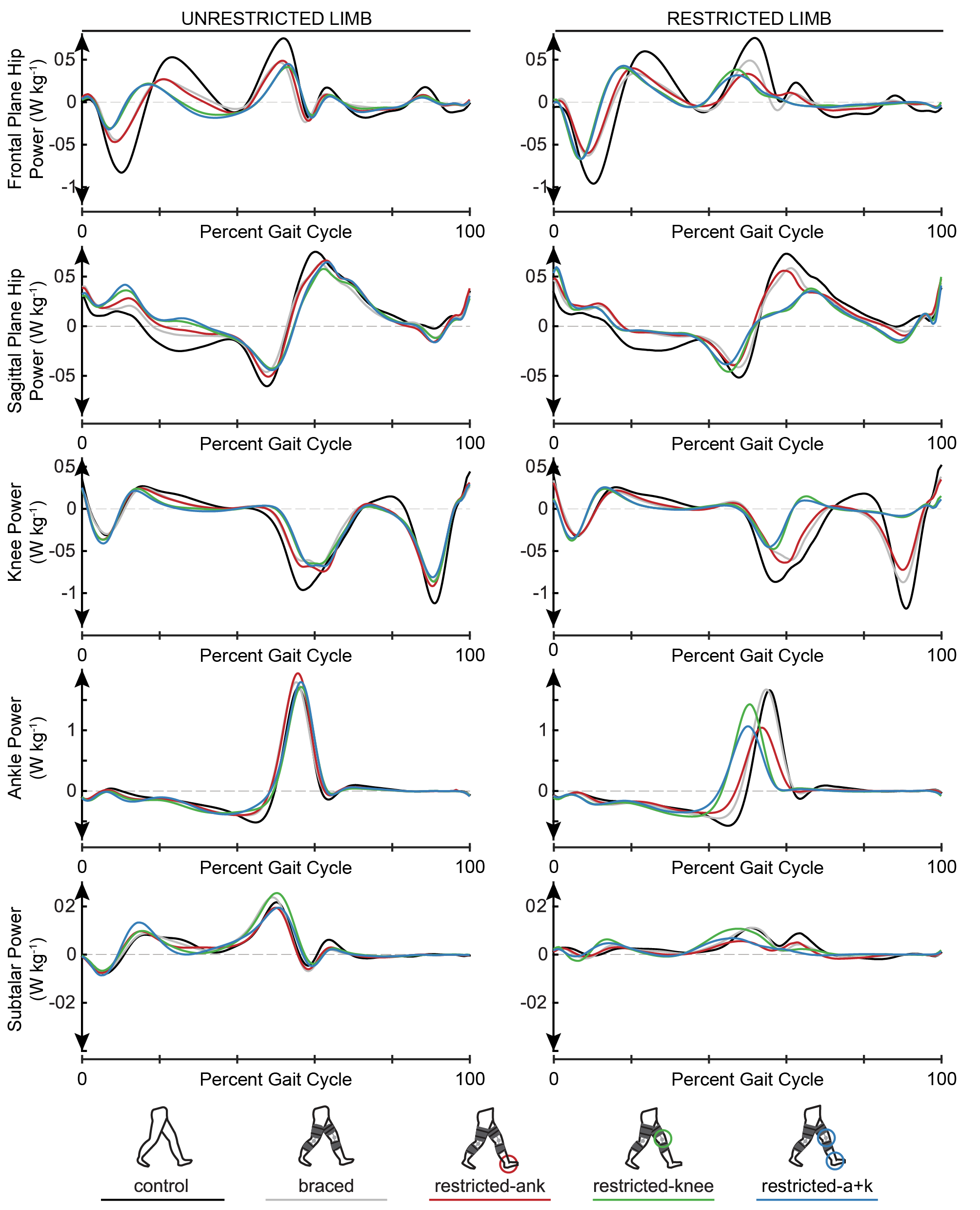

Supplement: Supplementary file 4 — Additional file 4: Fig. S4. Subject average joint powers. Subject average (N=15) joint powers for the control, braced and all restricted conditions for the unrestricted limb (left column) and the restricted limb (right column). [file 12984_2021_812_MOESM4_ESM.png]

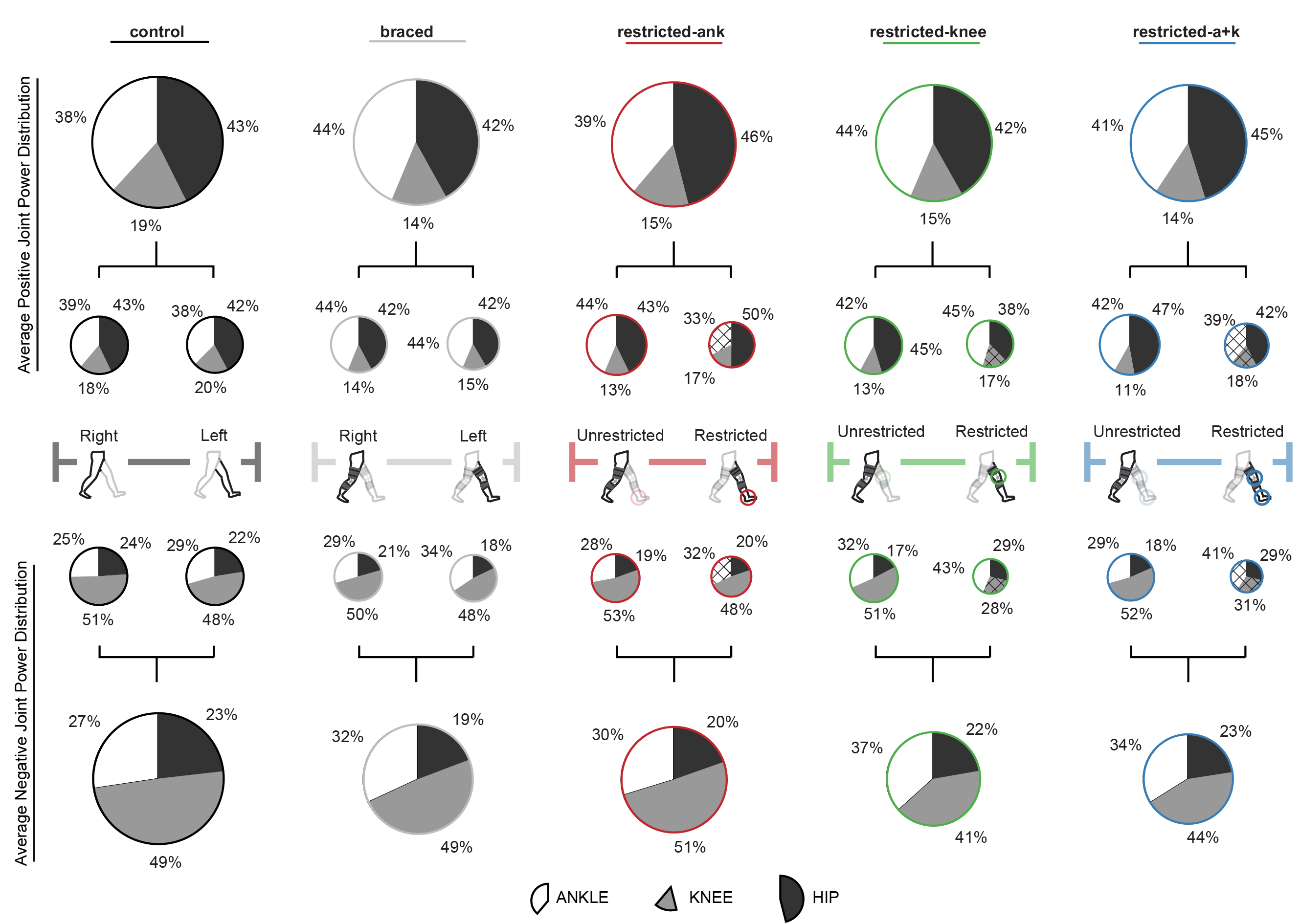

Supplement: Supplementary file 5 — Additional file 5: Fig. S5. Average positive (top) and negative (bottom) joint power distribution. Sections of pie chart represent the subject averaged (N=15) ankle (white), knee (light grey), and hip (dark grey) contributions and are organized by the following columns (from left to right): 1) control, braced, restricted-ank, restricted-knee, and restricted-a+k. Average positive joint powers were summed across both limbs in the top row, and average negative joint powers were summed across both limbs on the bottom row. The distribution of positive and negative power within each limb were indicated in the pie charts in the second and third row of the figure, respectively. Note that the diameters were scaled by dividing the sum of joint contributions for each pie by the maximum sum of average positive or negative joint powers (control) and that hatch patterns were used to indicate joints restricted. [file 12984_2021_812_MOESM5_ESM.png]
